# Bioinformatics analysis of the serine and glycine pathway in cancer cells

**DOI:** 10.18632/oncotarget.2668

**Published:** 2014-11-21

**Authors:** Alexey Antonov, Massimiliano Agostini, Maria Morello, Marilena Minieri, Gerry Melino, Ivano Amelio

**Affiliations:** ^1^ Medical Research Council, Toxicology Unit, Leicester University, Leicester LE1 9HN, UK; ^2^ Department of Experimental Medicine and Surgery, University of Rome “Tor Vergata”, Rome 00133, Italy; ^3^ Biochemistry Laboratory IDI-IRCC, University of Rome “Tor Vergata”, Rome 00133, Italy

**Keywords:** Cancer Metabolism, Serine, Glycine, survival analysis

## Abstract

Serine and glycine are amino acids that provide the essential precursors for the synthesis of proteins, nucleic acids and lipids. Employing 3 subsequent enzymes, phosphoglycerate dehydrogenase (PHGDH), phosphoserine phosphatase (PSPH), phosphoserine aminotransferase 1 (PSAT1), 3-phosphoglycerate from glycolysis can be converted in serine, which in turn can by converted in glycine by serine methyl transferase (SHMT). Besides proving precursors for macromolecules, serine/glycine biosynthesis is also required for the maintenance of cellular redox state. Therefore, this metabolic pathway has a pivotal role in proliferating cells, including cancer cells. In the last few years an emerging literature provides genetic and functional evidences that hyperactivation of serine/glycine biosynthetic pathway drives tumorigenesis. Here, we extend these observations performing a bioinformatics analysis using public cancer datasets. Our analysis highlighted the relevance of PHGDH and SHMT2 expression as prognostic factor for breast cancer, revealing a substantial ability of these enzymes to predict patient survival outcome. However analyzing patient datasets of lung cancer our analysis reveled that some other enzymes of the pathways, rather than PHGDH, might be associated to prognosis. Although these observations require further investigations they might suggest a selective requirement of some enzymes in specific cancer types, recommending more cautions in the development of novel translational opportunities and biomarker identification of human cancers.

## INTRODUCTION

Cancer cells exhibit metabolic changes, which enable the malignant cells to sustain cell growth and proliferation [1–3]. Indeed, Otto Warburg was the first to describe that cancer cells preferentially use aerobic glycolysis to produce energy [4–7]. However, in the last 10 years significant effort has been made to the characterization of the metabolic alteration in cancer cells [8, 9]. It is now clear that together with the Warburg effect, the malignant cells show also an increased flux through to the pentose phosphate pathway, high glutamine consumption, maintenance of redox status and increased lipids biosynthesis, which all tighter help is sustaining cell proliferation under metabolic, redox stress or hypoxia [10–12]. Moreover, an increased uptake of glycine and serine has been observed [13–15]. However, serine can be also synthetized within the cells. Indeed, the *de novo* serine synthesis pathways represent one of the most significant pathways derived from a branching route of glycolysis. Serine can then converted in glycine, which provides the carbon units to fuel the one-carbon metabolism [16, 17]. One-carbon metabolism represents a complex metabolite network that is based on the chemical reactions of folate compounds [18]. This pathway provides the one carbon unit required for the synthesis of proteins, lipids, nucleic acids and other cofactors. The one-carbon unit proceeds in a cyclical pathway from where they are transferred to other metabolic pathways. The importance of this metabolic pathway is underlined by the fact that antimetabolic (anti-folate) chemotherapy is currently widely employed in cancer treatment since its discovery more than 50 years ago [19–21]. It was in fact in 1947 when Sidney Farber at the Children's Hospital defined the use of antifolate therapy for leukemia, based on the work of the hematologists George Minot, who identified a critical micronutrient later defined as vitamin B12 (1934 Nobel Prize), and Lucy Willis, a clever physician from the London School of Medicine for Women who identified in Bombay a “Willis factor” from the popular yeastly spread “Marmite” which turned out to be folic acid.

In this perspective we will highlight the recent implication of serine and glycine metabolism in cancer biology. Recent reports indicate that the serine biosynthetic pathway is activated in cancer cells and represents an essential process in cancer pathogenesis [22]. Our bioinformatics analysis indicate that selective expression of some metabolic enzymes represents a prognostic factor for cancer, suggesting that activation of this metabolic pathway can be associated to the pathogenesis of different cancer types.

### Serine pathway in cancer cells

**Figure 1:**
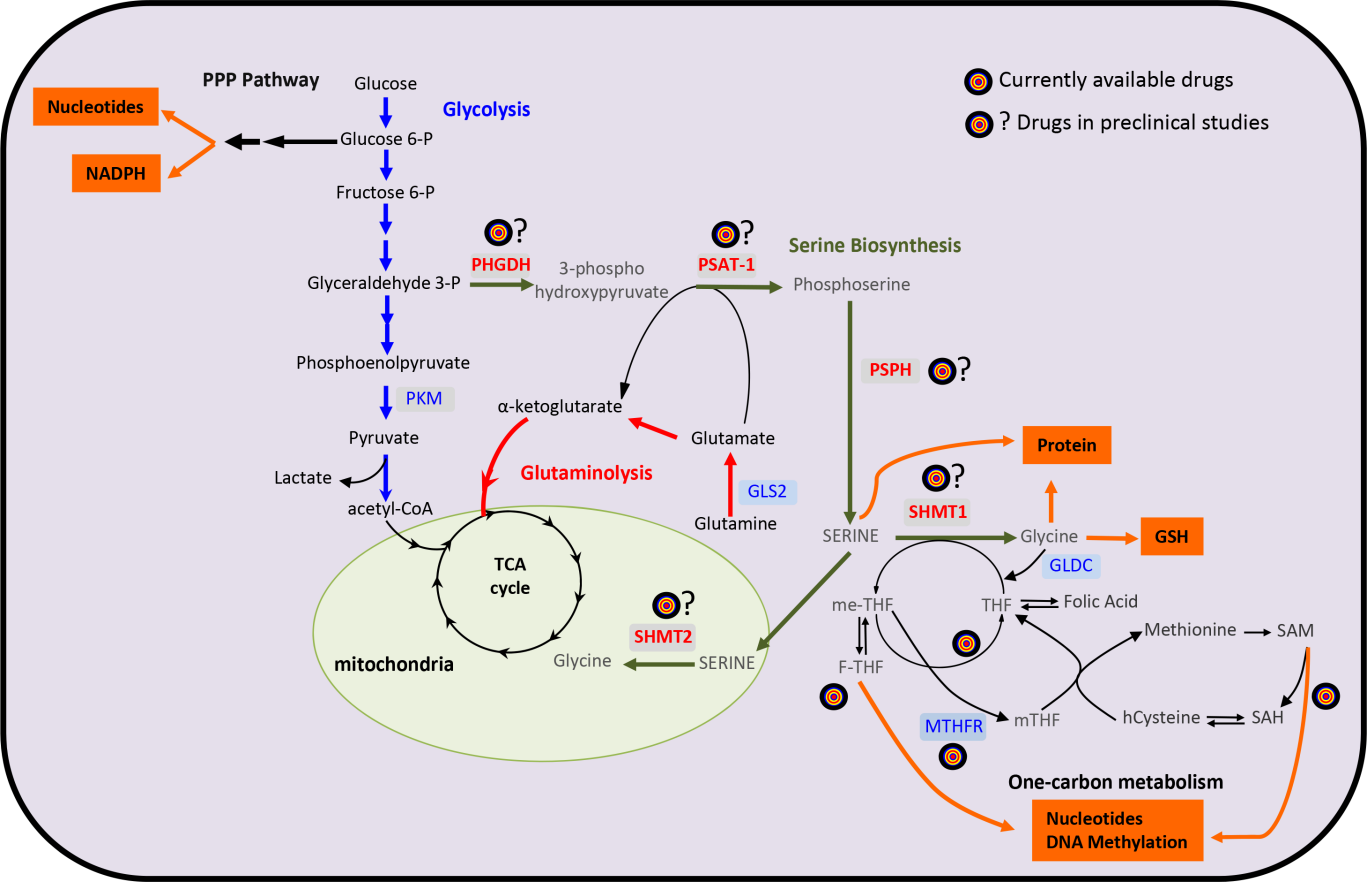
A schematic overview of the metabolic pathways involved in cancer biology Cancer cells show an increased flux through to the glycolysis, pentose phosphate pathway and high glutamine consumption. Moreover, they also show an increased uptake of glycine and serine. In particular, the serine synthesis pathway utilizes the glycolytic intermediate glycerate-3-phosphate, which is converted by PHGDH, PSAT-1 and PSPH into serine. De novo synthetized serine and glycine fuel on-carbon metabolism. The one-carbon metabolism plays an essential role in the generation of proteins, nucleotides, GSH and substrates for methylation reactions. In red are the cancer-associated genes. PHGDH, phosphoglycerate dehydrogenase; PSAT-1, phosphoserine aminotransferase 1; PSPH, phosphoserine phosphatase; SHMT, Serine hydroxymethyltransferase; GLS-2, glutaminase 2; GLDC, glycine decarboxylase; GSH, glutathione; MTHFR, methylenetetrahydrofolate reductase; SAM, *S*-adenosylmethionine; SAH, *S-*adenosylhomomocysteine; THF, tetrahydrofolate; me-THF, 5,10-methylenetetrahydrofolate; F-THF, 10-formyltetrahydrofolate; mTHF, 5-methyltetrahydrofolate; PKM, pyruvate kinase; PPP, pentose phosphate pathway.

Glucose and glutamine are two nutrients that cancer cells utilize for supporting energy metabolism and anabolic processes [23, 24]. However, cancer cells also increase de novo synthesis of serine and glycine that provides methyl group required for the biosynthetic pathways and DNA methylation. Indeed, the biosynthesis of serine and glycine was first found increased in lymphomas. In particular, it was shown, by radiolabeling experiments, that serine is formed prior glycine and that the glycolytic intermediate metabolite, 3-phosphoglycerate, is a common precursor [25]. In the last few years this early observation was also observed in breast cancer [26, 27] and melanoma [18]. Within the cells serine is synthetized by 3-phosphoglycerate through a 3-step enzymatic reaction. The first step of this metabolic pathway is the conversion of 3-phosphoglycerate in 3-phosphohydroxypyruvate, reaction catalyzed by phosphoglycerate dehydrogenase (PHGDH). Successively, 3-phosphohydroxypyruvate is converted in phosphoserine by the enzyme phosphoserine phosphatase (PSPH) and then in serine by phosphoserine aminotransferase 1 (PSAT1) (Figure 1) Serine can be also imported from the extracellular compartment by amino acid transporter.

The expression of PHGDH has been found upregulated (amplification of chromosome 1p12) in triple negative breast cancer and in melanoma, suggesting that tumors containing amplification of PHGDH take advantage from serine biosynthesis activity. Indeed, *in vitro* experiments show that inhibition of PHGDH expression induces a strong decrease in cell proliferation and a reduction in serine biosynthesis. Moreover, when PHDGH were overexpressed in the breast epithelial cells MCF10A (with no upregulated serine biosynthesis), the acinar morphology was disrupt and induces further phenotypic alterations that predispose to malignant transformation.

Oncogenic/oncosuppressor signalling can respond to nutrient stress and thus determine metabolic response in cancer cells. The tumour suppressor p53, beside the canonical response to DNA damage [28, 29] and control of cell cycle arrest [30–35] and apoptosis [36–48], plays a pivotal role in cellular metabolic homeostasis [49–51]. p53 helps cancer cells to face serine starvation, preserving cellular anti-oxidant capacity. Cells lacking p53 failed to respond to serine starvation, due to oxidative stress condition, which leads to reduced viability and severely impaired proliferation. During serine starvation, activation of p53-p21 axis leads to cell cycle arrest, which promotes cell survival by efficiently channeling depleted serine stores to glutathione synthesis [52–55]. The others p53-family members, with all the different expressed isoforms [56–58], determine a complex network which affect cellular metabolism [59]. TAp73 can control the balance of cellular metabolism [60–65], thus exerting its role in development and tumour suppression. TAp73 promotes serine/glycine biosynthetic pathway [66]. Similarly to p63 [67], p73 [68] promotes the expression of the glutaminase-2 (GLS-2), favoring glutaminolysis, which in turn pushes the second step of serine biosynthesis (Fig. 1). Overall, p53-family can influence various metabolic pathways, glycolysis, glutaminolosys, mitochondrial metabolism, fatty acids beta-oxidation etc., enabling cells to respond to metabolic stress [49, 63, 69–72]. However, p53 is mutated in about half of human cancers, resulting not only in a wt p53 loss-of-function but also a mutant p53 (mp53) gain-of-function (GOF) [73–77]. Part of mp53 GOF is due to mp53 interaction and consequent repression of the siblings, p63/p73 [78–81], making this scenario much more complex. Alteration of oncogenic/oncosupressor signaling might determine the expression of specific metabolic enzyme patterns, leading the cancer cells to rely on specific metabolic pathways.

**Figure 2:**
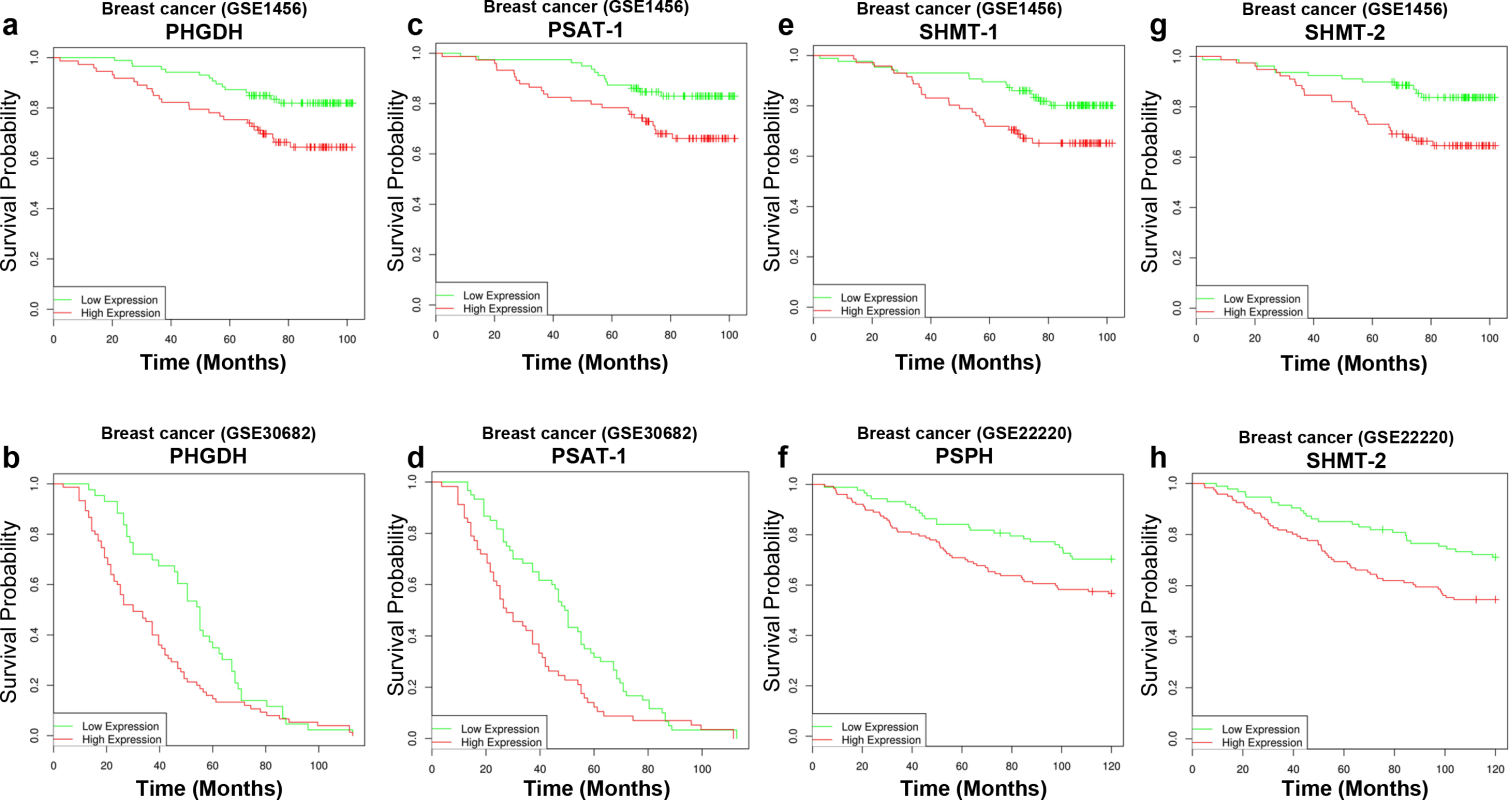
Survival analysis of serine/glycine biosynthetic enzymes Effect on survival outcome of the (PHGDH, PSAT-1, PSPH, SHMT-2). Clinical follow up data of different breast cancer datasets were censored for survival. Kaplan–Meier analysis with suppressed censoring showed a significant trend toward poor survival when serine biosynthetic enzymes were highly expressed.

In order to evaluate the importance of serine metabolic enzymes expression for cancer cells, we asked to search whether the enzymes of the serine metabolic pathway could function as prognostic marker [82, 83]. We assessed the clinical value of PHGDH in 17 breast and in 7 lung cancers human datasets. In 7 out of 17 breast cancer datasets high PHGDH expression represented a negative prognostic factor, predicting negative patient survival (Table 1, Fig. 2a,b). However, PHGDH did not appear to have any prognostic value in lung cancer datasets, whereas is some datasets PSAT-1, PSPH or SHMT-2 were able to predict patient survival (Table 1). This observation leads to the conclusion that although PHDGH seems to be involved in breast cancer pathogenesis, the alteration of serine biosynthesis might be still involved in other cancer types, employing different mechanisms.

**Table 1:** Survival outcome in human cancer datasets predicted by serine and glycine enzyme expression

*Cancer Type*	*GEO Dataset*	*PHGDH*	*PSAT-1*	*PSPH*	*SHMT-1*	*SHMT-2*
*Breast Cancer*	*GSE1456*	***Negative***	***Negative***	*Not sign*	***Negative***	***Negative***
*Breast Cancer*	*GSE3521*	*Not sign*	***Negative***	***Negative***	*Not sign*	***Negative***
*Breast Cancer*	*GSE30682*	***Negative***	***Negative***	*Not sign*	*Not sign*	*Not sign*
*Breast Cancer*	*GSE25055*	***Negative***	***Negative***	*Not sign*	*Not sign*	*Not sign*
*Breast Cancer*	*GSE22220*	*Not sign*	*Not sign*	***Negative***	*Not sign*	***Negative***
*Breast Cancer*	*GSE2034*	*Not sign*	*Not sign*	*Not sign*	*Not sign*	***Negative***
*Breast Cancer*	*GSE24450*	*Not sign*	***Negative***	*Not sign*	***Negative***	*Not sign*
*Breast Cancer*	*GSE3494*	***Negative***	*Not sign*	*Not sign*	*Not sign*	***Negative***
*Breast Cancer*	*GSE31448*	***Negative***	*Not sign*	*Not sign*	*Not sign*	***Negative***
*Breast Cancer*	*GSE21653*	***Negative***	*Not sign*	*Not sign*	***Positive***	***Negative***
*Breast Cancer*	*GSE25065*	***Negative***	***Negative***	*Not sign*	***Positive***	*Not sign*
*Breast Cancer*	*GSE11121*	*Not sign*	*Not sign*	*Not sign*	*Not sign*	***Negative***
*Breast Cancer*	*GSE19783*	*Not sign*	*Not sign*	*Not sign*	***Negative***	*Not sign*
*Breast Cancer*	*GSE17705*	*Not sign*	***Negative***	*Not sign*	*Not sign*	*Not sign*
*Breast Cancer*	*GSE22226*	*Not sign*	***Negative***	*Not sign*	***Positive***	*Not sign*
*Breast Cancer*	*GSE2990*	*Not sign*	*Not sign*	***Positive***	*Not sign*	***Negative***
*Breast Cancer*	*GSE7390*	*Not sign*	*Not sign*	*Not sign*	*Not sign*	*Not sign*
*Lung Cancer*	*GSE31210*	*Not sign*	***Negative***	*Not sign*	***Positive***	***Negative***
*Lung Cancer*	*GSE13213*	*Not sign*	***Negative***	***Positive***	*Not sign*	*Not sign*
*Lung Cancer*	*GSE19188*	*Not sign*	*Not sign*	***Negative***	*Not sign*	*Not sign*
*Lung Cancer*	*GSE36471*	*Not sign*	*Not sign*	*Not sign*	*Not sign*	*Not sign*
*Lung Cancer*	*GSE4882*	*Not sign*	*Not sign*	*Not sign*	*Not sign*	*Not sign*
*Lung Cancer*	*GSE11969*	*Not sign*	*Not sign*	*Not sign*	*Not sign*	*Not sign*
*Lung Cancer*	*GSE4573*	*Not sign*	*Not sign*	*Not sign*	*Not sign*	*Not sign*

### Glycine pathway in cancer cells

*De novo* synthesis of serine plays a crucial role as supplier of precursors for several biosynthetic pathways. Indeed, serine can be converted to glycine by the enzyme serine hydroxymethyltransferase (SHMT) [84, 85]. This reaction represents a major source of methyl groups for the one-carbon pools that are required for the biosynthesis of GSH, proteins, purines and DNA/histones methylation [86]. Therefore, SHMT occupies a critical position at the convergence of two key pathways for chemotherapeutic intervention: serine/glycine metabolism and nucleotide biosynthesis [87]. Within the cell, two isoforms of SHMT are present. SHMT1 is localized in the cytoplasm, whereas SHMT2 is present in the mithondria. Interestingly, c-Myc directly regulates the expression of both shmt1 and shmt2 genes [88–90]. More importantly, the expression and/or activity of the two enzymes are impaired in several tumors [91]. Several experimental evidences indicate that glycine uptake and catabolism can promote tumorigenesis, indicating that glycine metabolism could be a potential target for therapeutic intervention. Indeed, recently has been demonstrated that both glycine consumption and in particular, the expression of the mitochondrial glycine biosynthetic pathway correlate with the rate of proliferation across cancer cells [92–98]. This suggests, that under some circumstances, mitochondria play an essential role in supporting rapid cancer cell proliferation. In fact, inhibiting the expression of mitochondrial SHMT2 gene and deprivation of extracellular glycine were able to reduce HeLa cells proliferation, resulting in a prolonged G_1_ phase of the cell cycle. On the contrary, the upregulation of the serine/glycine metabolism was correlating with cell proliferation and poor prognosis in several tumors.

In our survival estimation analysis SHMT-2 resulted the most frequent significant prognostic factor among the serine/glycine biosynthetic enzymes. In 9 out of 17 breast cancer datasets high expression of SHMT-2 predicted negative prognosis (Table 1, Fig. 2c-h). On the other hand prediction ability of SHMT-1 appeared less clear. High expression of SHMT-1 indeed predicted in 3 datasets good prognosis and in 3 datasets negative prognosis, leaving very complex any conclusion (Table 1). From our analysis SHMT-2, even more than PHGDH, appears to be highly clinical relevant for breast cancer. Identification of selective SHMT-1/SHMT-2 selective inhibitors could be key for innovative and successful approaches.

## CONCLUSIONS AND PERSPECTIVES

Although the antimetabolites drugs were introduced in cancer therapy more than 50 years ago, they are still the most widely used drugs in cancer chemotherapy. Indeed, the antifolate agents were successfully used in the treatment of children affected by acute lymphoblastic leukemia. In the last decade many scientists have been attracted (or re-attracted) by the metabolic process associated with cancer biology. This boosted part of the scientific community to re-focus their effort in the development of novel antimetabolites drugs and/or in seeking new potential therapeutic targets (druggable metabolic enzymes) [99–105]. In fact, approved inhibitors of thymidylate and purine biosynthesis include methotrexate, pralatrexate, 5-fluorouracil and pemetrexed are currently in clinic. Among these, 5-fluorouracil is a standard agent for several cancer types, including colorectal cancer. The emerging role of serine/glycine/one-carbon metabolism in cancer biology opens the opportunity of alternative chemotherapeutic approaches. Indeed, mimicking uracil, 5-FU inhibits thymidine synthase, resulting in impairment of methylation of dUMP to dTMP and folate cycle disruption [106]. Several of these compounds are currently under pre-clinical evaluation or early-stage clinical trial. In addition, preclinical studies are currently ongoing also for small molecules targeting the catalytic site of metabolic enzymes, such as PHGDH PSAT, PSPH, GLDC [22]. Since the activity of metabolic enzymes can be modulated by the binding of an effector molecule at the allosteric site, it should be also considered to explore the possibility of generate small molecules that target allosteric binding site of the metabolic enzymes. However, our survival analysis (Table 1 and Fig. 2) highlighted that expression of some enzymes instead than others might be associated to the pathogenesis of different cancer types. This observation suggests more cautions. A selective drug targeting design for different cancer types could be critical to achieve therapeutic success. Therefore, it will be of importance to select subsets patients and to find the right combinations of chemical compounds targeting several metabolic enzymes of the serine/glycine pathway.

Pre-clinical and clinical studies have shown that reducing glucose intake was associated with negative effect on tumor growth [107–111]. Moreover, it has been show that in a tumor in xenograft mouse model, the tumor growth of p53n^−/−^ cells was reduced in mice fed with a diet containing no serine and glycine [52]. Overall, these observation indicate that an alternative therapeutic approach could be to associate with pharmacological agents including, a complementary diet or nutrient modification. However, it should be noticed that reduced intake of folate is also associated with breast and colorectal cancer, suggesting the complexity of the relationship between diet and one-carbon metabolism.

In conclusion, more work is needed in order to define the complexity of the metabolic pathways involved in cancer biology and the relationship between them. Moreover, we also need to understand not only the differences between normal and tumor cells, but also why some cancer cells are more dependent on specific metabolic pathways than others. This it could potentially improve the selectivity and the outcome of the therapy.
